# Proto-memformer: deformable memory transformer for Parkinson’s MRI classification

**DOI:** 10.1038/s41598-026-54050-w

**Published:** 2026-05-19

**Authors:** Ziyue Wang, Yisong Yao, Jia Chen

**Affiliations:** 1 Xiangya School of Nursing, Central South University, Changsha, China; 2 School of Nursing and Midwifery, University of Galway, Galway, Ireland; 3 Key Laboratory of Digital-Intelligent Disease Surveillance and Health Governance, North Sichuan Medical College, Nanchong, China

**Keywords:** Parkinson’s disease, MRI classification, Deformable attention, Prototype memory, Model robustness, Computational biology and bioinformatics, Engineering, Mathematics and computing, Neuroscience

## Abstract

This paper proposes a Prototype-Guided Deformable Memory Transformer (Proto-MemFormer) model for Parkinson’s Disease (PD) MRI classification. In the encoding stage, the model integrates a prototype-guided memory mechanism with a deformable attention structure to dynamically aggregate local morphological features and global semantic information. In the decoding stage, a position-calibrated retrieval module is introduced to enhance cross-sample feature alignment and discriminative representation. Experiments conducted on two public datasets, NTUA-Parkinson and PPMI, demonstrate that the proposed model achieves Accuracy, Precision, Recall, F1-Score, and AUC of 93.45%, 93.72%, 93.21%, 93.46%, and 94.81%, respectively, on the NTUA-Parkinson dataset, outperforming current state-of-the-art deep learning methods. Moreover, in the hyperparameter and training set scaling experiments, the model exhibits performance fluctuations of less than 3%, verifying its stability and robustness under different data and environmental conditions.

## Introduction

Parkinson’s Disease (PD) is a common neurodegenerative disorder, and its early diagnosis plays a critical role in patient intervention and disease progression control^1^. In recent years, with the advancement of Magnetic Resonance Imaging (MRI) technology, imaging-based computational diagnosis methods have gradually become an important auxiliary tool for clinical decision-making. Traditional Convolutional Neural Networks (CNNs) perform well in extracting local structural features but exhibit limited capability in modeling non-rigid anatomical variations and multi-scale semantic dependencies of the brain. This limitation constrains their ability to capture the global pathological representations^2^. Therefore, developing an efficient neuroimaging analysis model capable of simultaneously capturing local details and global semantics is of great significance for achieving more accurate PD identification^3^.

Despite the remarkable progress of deep learning-based visual models in medical image classification, existing approaches still face several challenges when applied to PD MRI data^4,5^. First, the spatial distribution of MRI features is sparse and exhibits complex deformations, making it difficult for conventional attention mechanisms to effectively capture inter-regional correlations. Second, the limited number of medical imaging samples often leads to model overfitting, resulting in unstable feature representations^6^. Third, variations in scanning conditions and acquisition centers may introduce feature shifts, weakening the discriminative performance of the model. These challenges collectively lead to suboptimal classification accuracy and robustness under complex imaging conditions.

To address the above issues, this paper proposes a Prototype-Guided Deformable Memory Transformer (Proto-MemFormer) model for PD MRI classification. The proposed method integrates a prototype-guided mechanism and deformable attention structure into the encoder to achieve multi-scale semantic aggregation and structural alignment. Specifically, the prototype memory provides cross-sample semantic anchors that suppress subject-specific noise and scanner-induced variability, while deformable attention learns content-adaptive offsets to establish flexible correspondences among anatomically mismatched regions, effectively accommodating non-rigid neuroanatomical variations. Meanwhile, a position-calibrated retrieval decoder is designed to perform cross-sample semantic reorganization and feature alignment, thereby enhancing adaptability to non-rigid morphological variations. Through an end-to-end optimization strategy, the model achieves a dynamic balance between local feature representation and global semantic discrimination.

The main innovations and contributions of this study are summarized as follows:

1. A dual-stage architecture combining prototype memory and deformable attention is proposed to capture both local morphological variations and global semantic correlations in PD MRI images.
2. A position-calibrated retrieval decoder is designed to perform cross-sample semantic aggregation and feature reorganization, improving the model’s robustness to complex brain structural variations.
3. Extensive experiments on two public datasets, NTUA-Parkinson and PPMI, demonstrate that the proposed model significantly outperforms existing state-of-the-art methods across multiple evaluation metrics.
4. Further analyses on hyperparameter sensitivity, experimental environments, and data scale confirm the model’s stability and generalization capability under different conditions, providing an efficient and interpretable solution for intelligent medical image diagnosis.

## Related work

### Representation learning for medical image analysis

In recent years, representation learning has received extensive attention in the field of medical image analysis^7,8^. Its core objective is to automatically extract discriminative and generalizable feature representations in a data-driven manner, thereby improving the performance of downstream tasks such as segmentation, classification, and detection. Litjens et al.^9^ systematically reviewed the progress of deep learning in medical image analysis and pointed out that convolutional neural networks can significantly enhance feature representation capabilities across different modalities. Subsequently, Getty et al.^10^ further integrated representation learning with neuromorphic computing frameworks, proposing a deep representation model capable of maintaining strong discriminative power under low-sample conditions. Mall et al.^11^ summarized recent advances in structural optimization and performance enhancement of deep neural networks for medical image processing, emphasizing the importance of multi-level feature fusion in complex pathological scenarios. In addition, Yousef et al.^12^ provided a holistic overview of deep learning applications in medical imaging, suggesting that cross-modal semantic alignment and hierarchical knowledge modeling should be incorporated into network design to achieve unified representations of multi-source medical data.

With the rapid development of self-supervised and transfer learning, researchers have begun to explore efficient representation learning methods that do not rely on large-scale annotated datasets. Huang et al.^13^ conducted a systematic study on self-supervised learning for medical image classification and proposed standardized implementation guidelines for robust feature learning. Tang et al.^14^ introduced an efficient 3D representation learning framework that effectively addresses the challenges of feature sparsity and limited memory in volumetric medical imaging. Chartsias et al.^15^ incorporated a disentangled representation mechanism to jointly model structural and semantic information in cardiac image analysis. Morid et al.^16^ and Pachetti et al.^17^ reviewed research progress from the perspectives of transfer learning and few-shot learning, respectively, providing new insights into data-efficient representation learning for medical imaging tasks. Moreover, Krishnapriya et al.^18^ conducted a brain MRI classification study based on pre-trained deep models, confirming the effectiveness of pretrained features in neuroimaging analysis. Collectively, these studies demonstrate that deep representation learning has become a crucial paradigm for advancing intelligent diagnosis and medical image understanding.

### Transformer-based frameworks in neuroimaging classification

In recent years, the Transformer architecture has demonstrated remarkable capability in feature modeling and contextual representation for neuroimaging classification tasks. Reddy et al.^19^ were among the first to apply a fine-tuned Vision Transformer to multi-class brain MRI classification, showing that the self-attention mechanism can capture complex non-local dependencies among anatomical structures. Subsequently, Alp et al.^20^ proposed a joint Transformer framework for feature interaction learning in 3D MRI, significantly improving classification accuracy in Alzheimer’s disease recognition. Furthermore, Alp et al.^21^ combined Vision Transformer with a temporal Transformer to construct a multi-scale spatiotemporal encoding framework, providing a new direction for longitudinal MRI sequence analysis. Zhao et al.^22^ enhanced 3D MRI structural representation and automated discrimination by integrating convolutional features with Transformer-based attention. In addition, Alharthi et al.^23^ systematically reviewed the multimodal applications of Transformer models in autism spectrum disorder imaging, highlighting their potential for cross-modal transfer learning.

Cong et al.^24^ provided a comprehensive review of the development of Transformer models in brain imaging analysis from the perspectives of neuroscience, neurology, and psychiatry, emphasizing their strengths in cross-domain feature alignment and interpretable modeling. Ahmed et al.^25^ integrated Vision Transformer with GRU and proposed a hybrid sequential modeling framework for brain tumor detection, achieving dynamic fusion of spatial and temporal features. Dhinagar et al.^26^ introduced a Masked Autoencoder-based Transformer framework for parameter-efficient fine-tuning, effectively reducing computational costs in neuroimaging analysis. Collectively, these studies indicate that the Transformer architecture has become a mainstream paradigm in neuroimaging classification, providing structurally robust and cross-domain generalizable representations for various brain disorders, including Parkinson’s disease, and offering theoretical and practical foundations for the proposed Proto-MemFormer model.

## Method

### Overall model architecture

**Figure 1:**
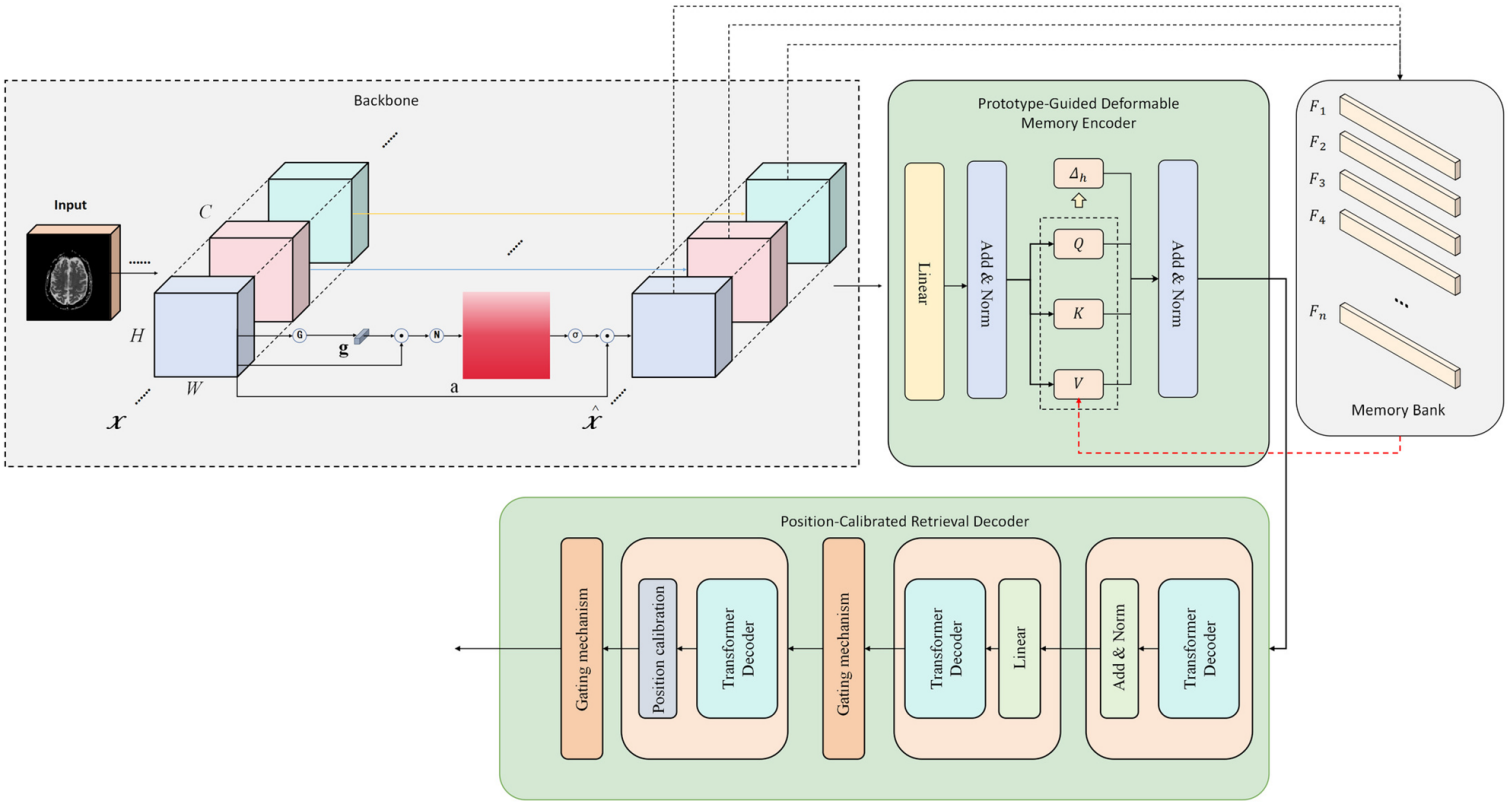
Overall architecture of the proposed Proto-MemFormer model. The network consists of a prototype-guided deformable memory encoder and a position-calibrated retrieval decoder, which collaboratively extract and align multiscale lesion features from MRI images for Parkinson’s disease classification.

The proposed Proto-MemFormer model adopts an encoder–decoder architecture, introducing a prototype-guided deformable memory mechanism to establish a dynamic balance between global semantic alignment and local feature reconstruction. In particular, the prototype memory serves as a shared semantic reference across subjects, while deformable attention enables content-adaptive spatial correspondence under non-rigid neuroanatomical deformations, jointly supporting robust structural alignment and discriminative representation learning. The overall model architecture is shown in Figure 1.

The model takes two-dimensional brain MRI images as input, which are first processed through a convolutional embedding layer for spatial downsampling and channel expansion, mapping the input into a sequence of patch tokens. A lightweight memory bank $\mathscr {M}=\{m_1,m_2,\dots ,m_K\}$ is then introduced as a global prototype prior to capture stable feature patterns across samples. For the current sample representation $\textbf{X}\in \mathbb {R}^{N\times C}$, it is first projected into the prototype space through a linear bottleneck:

1 $$\begin{aligned} \textbf{Z} = \phi (\textbf{X}) = \textbf{X}\textbf{W}_p + \textbf{b}_p , \end{aligned}$$

where $\phi (\cdot )$ denotes the MLP projection, and $\textbf{W}_p$ and $\textbf{b}_p$ are learnable parameters. Subsequently, a deformable multi-head self-attention mechanism (MH-DeSA) performs feature aggregation on content-dependent offset points, aligning the queries $\textbf{Q}$, keys $\textbf{K}$, and values $\textbf{V}$ with the global prototypes $\mathscr {M}$, thereby enabling dynamic reconstruction in the latent space:

2 $$\begin{aligned} \text {DeSA}(\textbf{Q},\textbf{K},\textbf{V},\mathscr {M}) = \sum _{h=1}^{H} \text {Softmax}\!\left( \frac{(\textbf{Q}_h+\Delta _h)\textbf{K}_h^\top }{\sqrt{d}}\right) \textbf{V}_h , \end{aligned}$$

where $\Delta _h$ denotes the content-dependent learnable offset, *H* is the number of attention heads, and *d* is the scaling factor. This process adaptively models non-rigid spatial variations and enhances the stability of structural representations among individuals within an implicit global semantic framework. The token sequence is then processed through a one-dimensional convolutional layer for local sequential modeling, which suppresses redundant tokens while preserving high-frequency textures and edge-related statistical information, providing structured inputs for subsequent routing and decoding.

In the decoding stage, a position-calibrated retrieval-based cross-attention mechanism (MH-DeCA) is employed to align and aggregate multi-level encoded features through a retrievable feature queue. For the decoding query $\textbf{q}$ and the feature keys and values $\textbf{K}_d,\textbf{V}_d$, the deformable cross-attention process is defined as:

3 $$\begin{aligned} \text {DeCA}(\textbf{q},\textbf{K}_d,\textbf{V}_d) = \text {Softmax}\!\left( \frac{(\textbf{q}+\Delta _p)\textbf{K}_d^\top }{\sqrt{d}}\right) \textbf{V}_d , \end{aligned}$$

where $\Delta _p$ represents the position offset calibration vector. After cross-attention aggregation, the output features are refined through an MLP-based channel reorganization and nonlinear gating to restore the discriminative dimensions diluted by attention. Finally, a linear layer followed by a Softmax head generates classification probabilities, distinguishing MRI scans of Parkinson’s patients from those of healthy individuals. The overall framework, while maintaining computational efficiency, integrates the three key components–prototype prior, deformable alignment, and position-aware retrieval–enabling robust discrimination for data characterized by subtle morphological differences and significant distribution shifts.

### Prototype-guided deformable memory encoder

**Figure 2:**
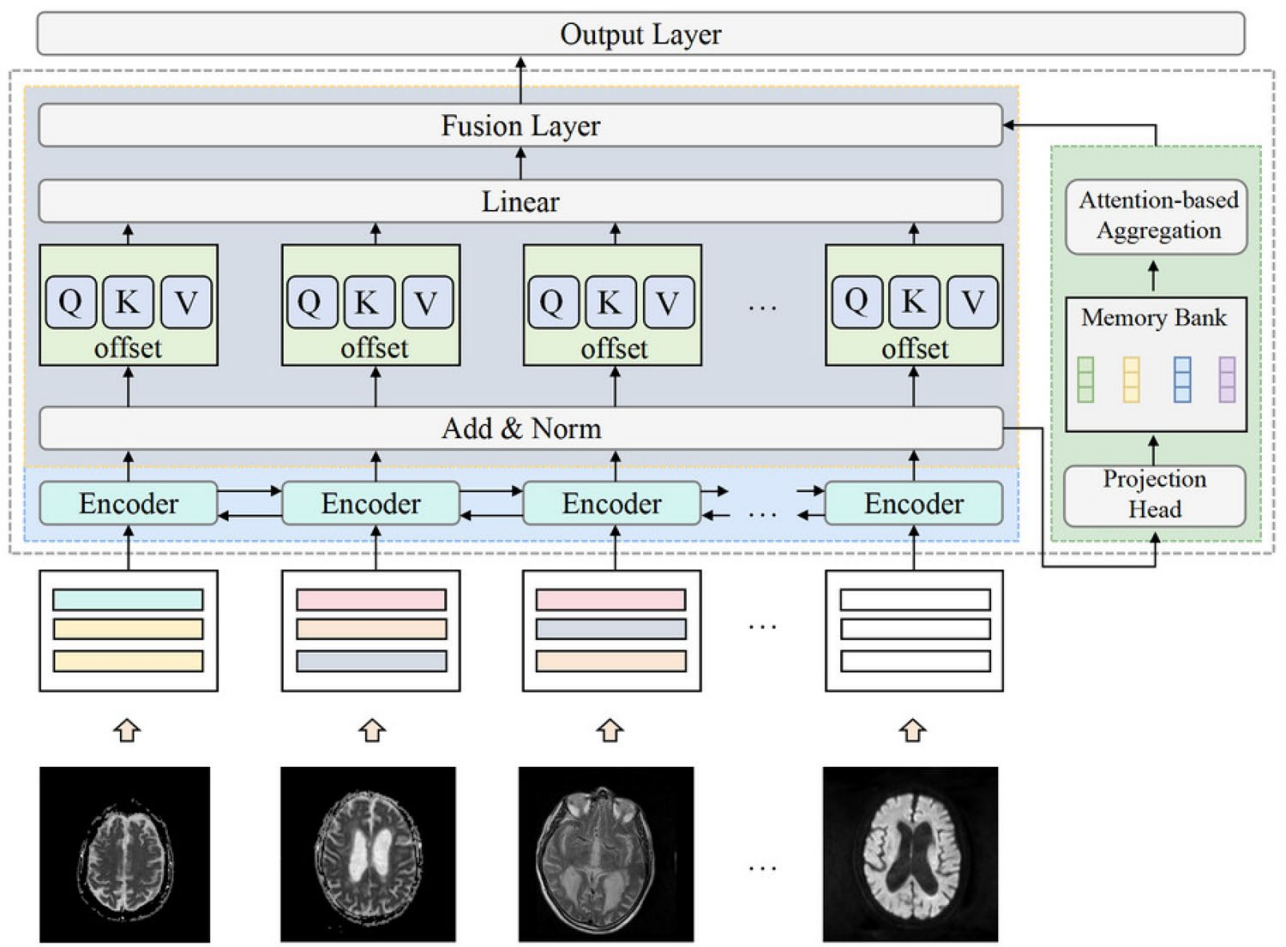
The proposed encoder architecture integrates a prototype-guided projection head and a deformable attention mechanism to extract multi-level semantic representations from MRI slices. Shared memory prototypes and attention-based aggregation further enhance feature alignment across subjects, enabling robust structural modeling for downstream classification.

The core objective of the encoder is to achieve globally consistent semantic representation across samples and input-adaptive deformable alignment. To this end, the proposed Prototype-Guided Deformable Memory Encoder integrates a prototype memory bank with a deformable multi-head self-attention mechanism, enabling the input features to be guided by global prototypes while dynamically adjusting their sampling patterns according to local structural variations. The module architecture is illustrated in Figure 2. Here, the “deformable memory” refers to a set of learnable prototype tokens that are queried by input-dependent attention with learned offsets, such that the effective correspondence between subject-specific anatomical patterns and global semantic anchors can be adaptively established.

In the overall process, the input MRI image is first mapped by a convolutional embedding layer into a sequence of patch tokens $\textbf{X}=\{\textbf{x}_1,\textbf{x}_2,\dots ,\textbf{x}_N\}\in \mathbb {R}^{N\times C}$, where *N* denotes the number of tokens and *C* represents the channel dimension. Each token $\textbf{x}_i\in \mathbb {R}^{C}$ corresponds to a local MRI patch, and $\textbf{X}$ serves as the encoder input sequence for subsequent prototype querying and deformable aggregation. To enhance distributional robustness, a learnable global prototype set $\mathscr {M}=\{m_1,m_2,\dots ,m_K\}\in \mathbb {R}^{K\times C}$ is constructed to provide prior anchors in the latent space. In practice, $\mathscr {M}$ is implemented as a bank of *K* trainable memory/prototype tokens, initialized randomly and updated jointly with the network parameters via back-propagation, so that each $m_k\in \mathbb {R}^{C}$ gradually captures a stable cross-subject semantic pattern (e.g., common structural configurations) in the training data. Each token is linearly projected into the prototype space as:

4 $$\begin{aligned} \textbf{z}_i = \phi (\textbf{x}_i) = \textbf{W}_p \textbf{x}_i + \textbf{b}_p, \quad i=1,\dots ,N , \end{aligned}$$

where $\textbf{W}_p\in \mathbb {R}^{C\times D}$ is the projection matrix and $\textbf{b}_p$ is the bias term. Here, $\phi (\cdot )$ denotes the prototype projection head that maps $\textbf{x}_i$ from the token space (*C* channels) to a compact prototype space of dimension *D* for similarity-based querying. The projected feature $\textbf{z}_i$ forms global guidance weights based on its similarity to the prototypes:

5 $$\begin{aligned} \alpha _{ik} = \frac{\exp (\textbf{z}_i^\top m_k / \tau )}{\sum _{j=1}^{K} \exp (\textbf{z}_i^\top m_j / \tau )} , \end{aligned}$$

where $\tau$ denotes the temperature parameter. $\alpha _{ik}$ denotes the soft assignment weight between token *i* and prototype *k*, and $\tau$ controls the sharpness of the assignment (smaller $\tau$ yields more peaked prototype selection). The prototype-weighted prior embedding is thus obtained as:

6 $$\begin{aligned} \tilde{\textbf{x}}_i = \sum _{k=1}^{K} \alpha _{ik} m_k . \end{aligned}$$

The sequence $\tilde{\textbf{X}}=\{\tilde{\textbf{x}}_1,\dots ,\tilde{\textbf{x}}_N\}$ can be viewed as a prototype-guided representation, where each token is “pulled” toward a cross-subject semantic anchor to reduce subject-specific noise and scanner-induced intensity/texture variations.

To achieve deformable structural alignment at the spatial level, a multi-head self-attention mechanism with offset learning (MH-DeSA) is introduced. For each attention head *h*, the query, key, and value matrices are defined as:

7 $$\begin{aligned} \textbf{Q}_h = \textbf{X}\textbf{W}_h^Q, \quad \textbf{K}_h = \textbf{X}\textbf{W}_h^K, \quad \textbf{V}_h = \textbf{X}\textbf{W}_h^V , \end{aligned}$$

where $\textbf{W}_h^Q,\textbf{W}_h^K,\textbf{W}_h^V\in \mathbb {R}^{C\times d_h}$ are the linear projection parameters. Here, $\textbf{Q}_h,\textbf{K}_h,\textbf{V}_h\in \mathbb {R}^{N\times d_h}$ denote the head-specific query/key/value features, $d_h$ is the per-head channel dimension, and *H* is the number of heads. The deformable offset $\Delta _h$ is learned through a content-dependent network $\mathscr {F}_\theta$:

8 $$\begin{aligned} \Delta _h = \mathscr {F}_\theta (\textbf{Q}_h, \tilde{\textbf{X}}), \end{aligned}$$

where $\tilde{\textbf{X}}$ denotes the prototype-guided feature sequence. $\mathscr {F}_\theta (\cdot )$ is implemented as a lightweight MLP that predicts token-wise offsets conditioned on the current subject’s content (via $\textbf{Q}_h$) and the prototype-guided anchors (via $\tilde{\textbf{X}}$), so that $\Delta _h\in \mathbb {R}^{N\times d_h}$ becomes subject-adaptive and can compensate for non-rigid anatomical misalignment across subjects. The attention weights with learned offsets are formulated as:

9 $$\begin{aligned} \textbf{A}_h = \text {Softmax}\!\left( \frac{(\textbf{Q}_h+\Delta _h)(\textbf{K}_h+\Delta _h)^\top }{\sqrt{d_h}}\right) . \end{aligned}$$

$\textbf{A}_h\in \mathbb {R}^{N\times N}$ denotes the normalized attention map, and adding $\Delta _h$ effectively perturbs the matching geometry in a content-driven manner, allowing the model to establish flexible correspondences between anatomically mismatched regions (e.g., slight shifts/asymmetries) instead of enforcing fixed token-to-token alignment. The final deformable attention output is obtained as:

10 $$\begin{aligned} \textbf{Y}_h = \textbf{A}_h \textbf{V}_h, \quad \textbf{Y} = \text {Concat}(\textbf{Y}_1,\dots ,\textbf{Y}_H)\textbf{W}^O , \end{aligned}$$

where $\textbf{W}^O\in \mathbb {R}^{Hd_h\times C}$ is the output projection matrix. $\textbf{Y}_h\in \mathbb {R}^{N\times d_h}$ is the head output, $\textbf{Y}\in \mathbb {R}^{N\times C}$ is the fused representation, and $\textbf{W}^O$ projects the concatenated heads back to *C* channels. The multi-head aggregation adaptively models multi-scale features, allowing the model to allocate attention dynamically to different spatial locations according to the structural characteristics of the input, thereby capturing subtle morphological variations. Compared with fixed attention (or fixed memory tokens), the learned offsets enable head-wise adaptivity to subject-specific anatomical variations, which is crucial for PD MRI where discriminative patterns may appear as subtle and spatially non-uniform morphological deviations across individuals.

On the aggregated sequence, a one-dimensional convolution module is further applied as a “token router” to introduce local structural inductive bias, strengthening sequential dependencies and local statistical features. This process is formalized as:

11 $$\begin{aligned} \textbf{R} = \sigma (\text {Conv1D}(\textbf{Y})) + \textbf{Y}, \end{aligned}$$

where $\sigma (\cdot )$ denotes a nonlinear activation function. Here, $\text {Conv1D}(\cdot )$ operates along the token dimension to emphasize locally consistent patterns and suppress redundant responses, and the residual connection preserves global contextual cues from deformable attention. The output $\textbf{R}$ represents the context representation refined through prototype guidance and deformable aggregation, maintaining global consistency across samples while preserving spatial variability at the individual level. $\textbf{R}\in \mathbb {R}^{N\times C}$ is the final encoder output that will be passed to the retrieval-based decoder, serving as the local transformer representation augmented by prototype-aligned memory guidance. This encoder design enables the model to stably extract high-dimensional discriminative features even under conditions where MRI differences between Parkinson’s patients and healthy individuals are extremely subtle, thus providing structured semantic support for the subsequent retrieval-based decoder.

### Position-calibrated retrieval decoder

**Figure 3:**
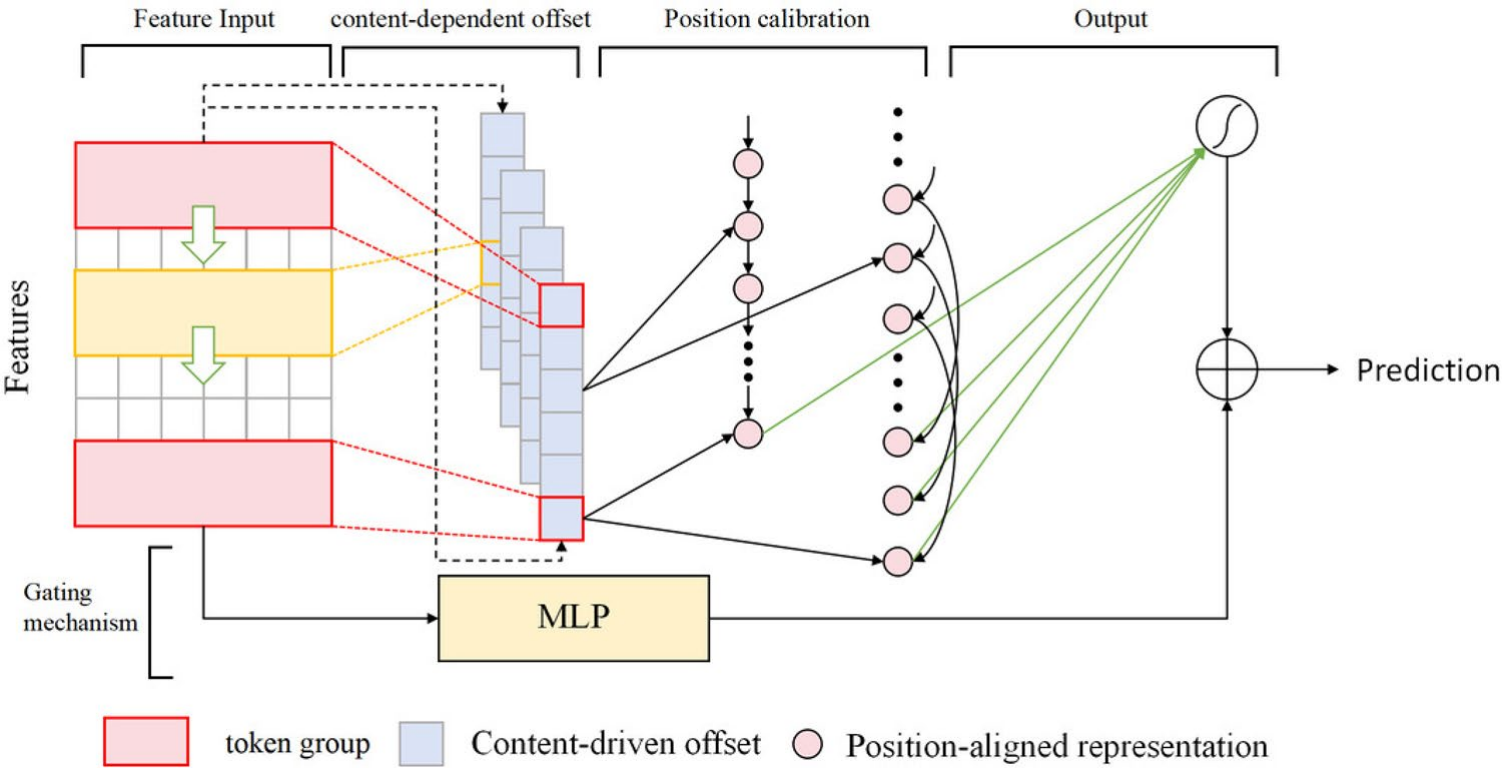
The Position-Calibrated Retrieval Decoder integrates content-driven offsets with a position calibration module to generate structure-aligned representations from encoder features. By combining deformable retrieval, channel-wise gating, and calibrated fusion, the decoder produces robust semantics for final prediction.

After the encoder completes multi-scale prototype guidance and deformable aggregation, the obtained contextual representation $\textbf{R}=\{\textbf{r}_1,\textbf{r}_2,\dots ,\textbf{r}_N\}$ still contains redundant spatial information and imbalanced semantic distributions. Here, $\textbf{R}\in \mathbb {R}^{N\times C}$ denotes the encoder output token sequence, where each $\textbf{r}_i\in \mathbb {R}^{C}$ corresponds to a patch-level contextual feature of the current 2D MRI slice. To address this, a Position-Calibrated Retrieval Decoder (PCRD) is proposed, which employs a retrievable feature queue as the medium and performs cross-attention-based semantic reorganization and positional alignment at both spatial and channel levels. The key idea is to retrieve cross-token evidence from a structured queue built from $\textbf{R}$, and then fuse the retrieved evidence with local transformer representations through gating and calibrated aggregation. The module architecture is illustrated in Figure 3.

Specifically, a feature memory queue $\mathscr {K}_d=\{\textbf{k}_1,\dots ,\textbf{k}_L\}$ and $\mathscr {V}_d=\{\textbf{v}_1,\dots ,\textbf{v}_L\}$ is first constructed, where multi-level feature maps from the encoder are linearly compressed as:

12 $$\begin{aligned} \textbf{k}_l = \textbf{W}_k \textbf{r}_l + \textbf{b}_k, \quad \textbf{v}_l = \textbf{W}_v \textbf{r}_l + \textbf{b}_v, \quad l=1,\dots ,L, \end{aligned}$$

where $\textbf{W}_k,\textbf{W}_v\in \mathbb {R}^{C\times D}$ denote the projection matrices. In implementation, $\mathscr {K}_d$ and $\mathscr {V}_d$ are constructed deterministically from the encoder outputs of the current sample by projecting $\textbf{R}$ into a shared retrieval space of dimension *D*; *L* denotes the total number of retrievable items (e.g., $L=N$ when using a single-level token queue, or larger when concatenating multi-level encoder tokens). Meanwhile, a set of query tokens $\textbf{Q}=\{\textbf{q}_1,\dots ,\textbf{q}_T\}\in \mathbb {R}^{T\times D}$ is initialized, whose initial positions are generated from the global averaged feature $\bar{\textbf{r}}=\frac{1}{N}\sum _i \textbf{r}_i$ as:

13 $$\begin{aligned} \textbf{q}_t = \bar{\textbf{r}}\textbf{W}_q + \textbf{p}_t, \quad t=1,\dots ,T, \end{aligned}$$

where $\textbf{p}_t$ represents the learnable positional encoding. Here, *T* is the number of decoder queries, $\bar{\textbf{r}}\in \mathbb {R}^{C}$ provides a global slice-level summary, $\textbf{W}_q$ maps it to the retrieval space, and $\textbf{p}_t$ encodes the initial query position to maintain spatial sensitivity. The decoder first computes the cross-attention retrieval weights:

14 $$\begin{aligned} \textbf{A} = \text {Softmax}\!\left( \frac{(\textbf{Q}+\Delta _p)(\mathscr {K}_d)^\top }{\sqrt{D}}\right) , \end{aligned}$$

where $\Delta _p=\mathscr {G}_\theta (\textbf{Q})$ denotes the content-generated deformable offset and $\mathscr {G}_\theta (\cdot )$ represents a lightweight offset network. $\Delta _p\in \mathbb {R}^{T\times D}$ is predicted for each query token and makes the retrieval geometry content-adaptive; therefore, different subjects can shift their retrieval focus toward anatomically corresponding but spatially mismatched regions, which is essential for accommodating subject-specific non-rigid variations. Based on these weights, structured retrieval aggregation is performed as:

15 $$\begin{aligned} \tilde{\textbf{Q}} = \textbf{A}\mathscr {V}_d , \end{aligned}$$

which is equivalent to selecting locally relevant evidence from the feature memory $\mathscr {V}_d$ for secondary spatial aggregation and semantic alignment. Here, $\tilde{\textbf{Q}}\in \mathbb {R}^{T\times D}$ can be viewed as the retrieved memory-enhanced representation, while $\textbf{A}\in \mathbb {R}^{T\times L}$ explicitly specifies how each query token aggregates evidence from the queue.

To further suppress channel ambiguity caused by attention dilution, a channel reorganization module is introduced to perform nonlinear remapping of the aggregated features. It is defined as:

16 $$\begin{aligned} \textbf{Z} = \sigma (\tilde{\textbf{Q}}\textbf{W}_1 + \textbf{b}_1)\textbf{W}_2 + \textbf{b}_2, \end{aligned}$$

where $\sigma (\cdot )$ denotes the activation function, and $\textbf{W}_1,\textbf{W}_2\in \mathbb {R}^{D\times D}$ are learnable parameters. This module acts as a feature fusion and gating operator in the channel space, reshaping the retrieved evidence to emphasize discriminative dimensions and to reduce noise introduced by scanner differences or subtle texture variations. This bilinear mapping acts as a gating control and feature redistribution mechanism along the channel dimension, amplifying the discriminative features at the output stage. Considering spatial consistency, a positional calibration term is introduced to achieve geometric adjustment of the output:

17 $$\begin{aligned} \hat{\textbf{Z}}_t = \textbf{Z}_t + \Psi (\textbf{p}_t,\Delta _p), \end{aligned}$$

where $\Psi (\cdot )$ denotes a calibration function that remaps the output according to the relative positions associated with the offset $\Delta _p$. $\Psi (\textbf{p}_t,\Delta _p)$ provides an explicit position-aware correction term, ensuring that the retrieved features remain comparable across different scanning positions and alignment states. This mechanism can be regarded as a reverse compensation of the encoder offsets, ensuring discriminative robustness across different scanning positions and alignment states.

During the fusion stage, multi-head retrieval results are aggregated into a global representation using a weighted strategy:

18 $$\begin{aligned} \textbf{H} = \sum _{h=1}^{H} \omega _h \hat{\textbf{Z}}_h, \end{aligned}$$

where $\omega _h$ denotes the adaptive weighting coefficients. Here, $\omega _h$ is produced by a lightweight gating function (Softmax over head-wise pooled responses), enabling the model to adaptively fuse local and retrieved evidence from different heads according to subject-specific structural patterns. Finally, a linear layer maps the global representation into the category space:

19 $$\begin{aligned} \textbf{y} = \text {Softmax}(\textbf{H}\textbf{W}_c + \textbf{b}_c), \end{aligned}$$

where $\textbf{W}_c\in \mathbb {R}^{D\times 2}$ corresponds to the binary classification output. $\textbf{y}\in \mathbb {R}^{2}$ denotes the predicted class probability for PD vs. healthy controls, and $\textbf{W}_c,\textbf{b}_c$ are learnable classifier parameters. Through the proposed retrieval–calibration–reorganization decoding path, the model achieves focused information aggregation and positional consistency without relying on explicit structural differences. Complementing the encoder, the PCRD transforms deformable spatial representations from the encoding phase into high-confidence retrieval of discriminative cues, thereby enabling robust cross-sample feature fusion and globally consistent decision-making. In summary, the decoder fuses (i) local contextual representations produced by the encoder and (ii) retrieved memory evidence selected by deformable cross-attention, which improves discriminative power when PD-related structural differences are subtle and spatially non-uniform across subjects.

### Training objective

During the training phase, the encoder and decoder are jointly optimized to achieve semantic consistency from local representation to global discrimination. The encoder constructs a dynamic semantic space of input features through prototype guidance and deformable aggregation, while the decoder extracts high-confidence global semantic evidence based on the position-calibrated retrieval mechanism. The interaction between the two is reflected in the following manner: the encoder provides memory representations with structural awareness and cross-sample alignment capability, and the decoder performs semantic reorganization and evidence fusion on this basis, balancing spatial variability and channel discriminability within the latent space. This interaction ensures that the model maintains stable representational consistency and discriminative robustness under large variations in input distributions. The overall end-to-end training procedure is summarized in Algorithm 1.

**Algorithm 1:**
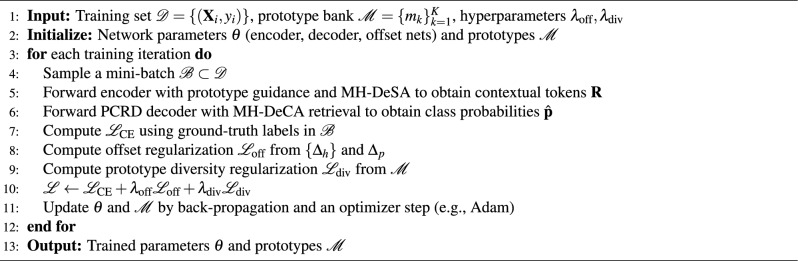
End-to-end training of Proto-MemFormer

The overall optimization objective adopts the standard cross-entropy loss to minimize the divergence between the predicted probability distribution and the ground truth label distribution. For a given sample set $\{(\textbf{X}_i, y_i)\}_{i=1}^{M}$, the model outputs the predicted probability $\hat{\textbf{p}}_i = f_{\theta }(\textbf{X}_i)$, where $f_{\theta }(\cdot )$ denotes the entire encoder–decoder interactive network. The cross-entropy loss is defined as:

20 $$\begin{aligned} \mathscr {L}_{\text {CE}} = -\frac{1}{M} \sum _{i=1}^{M} \sum _{c=1}^{C} \textbf{1}(y_i=c) \log \hat{p}_{i,c}, \end{aligned}$$

where $C=2$ corresponds to the two categories of Parkinson’s disease and healthy subjects, $\textbf{1}(\cdot )$ is the indicator function, and $\hat{p}_{i,c}$ represents the predicted probability that the *i*-th sample belongs to class *c*. All learnable components, including the encoder, the PCRD decoder, the prototype memory bank $\mathscr {M}$, and the offset networks (e.g., $\mathscr {F}_\theta$ and $\mathscr {G}_\theta$), are updated jointly via back-propagation under $\mathscr {L}_{\text {CE}}$. To stabilize the learning of deformable offsets and avoid excessive deformations, we additionally impose an offset magnitude regularization term

21 $$\begin{aligned} \mathscr {L}_{\text {off}} = \frac{1}{H N}\sum _{h=1}^{H}\sum _{i=1}^{N} \left\Vert \Delta _h(i)\right\Vert _2^2 \; + \; \frac{1}{T}\sum _{t=1}^{T}\left\Vert \Delta _p(t)\right\Vert _2^2, \end{aligned}$$

where $\Delta _h(i)$ denotes the offset vector associated with token *i* at head *h* in MH-DeSA, and $\Delta _p(t)$ denotes the offset vector of query token *t* in MH-DeCA. We further encourage prototype diversity to reduce memory collapse by applying $\ell _2$-normalization to each prototype token and adding a lightweight decorrelation penalty

22 $$\begin{aligned} \mathscr {L}_{\text {div}} = \frac{1}{K(K-1)} \sum _{k\ne j} \left( \frac{m_k^\top m_j}{\Vert m_k\Vert _2 \Vert m_j\Vert _2} \right) ^2. \end{aligned}$$

The final objective is therefore

23 $$\begin{aligned} \mathscr {L} = \mathscr {L}_{\text {CE}} + \lambda _{\text {off}}\mathscr {L}_{\text {off}} + \lambda _{\text {div}}\mathscr {L}_{\text {div}}, \end{aligned}$$

where $\lambda _{\text {off}}$ and $\lambda _{\text {div}}$ control the trade-off between classification supervision, offset stability, and prototype diversity. This loss function drives end-to-end joint updates of the encoder and decoder under semantic consistency, enabling the global prototype guidance and local retrieval aggregation to achieve optimal synergy in the discriminative space, thereby ensuring stable and reliable classification performance.

## Datasets and evaluation metrics

### Datasets

#### NTUA-prakinson-dataset

**Figure 4:**
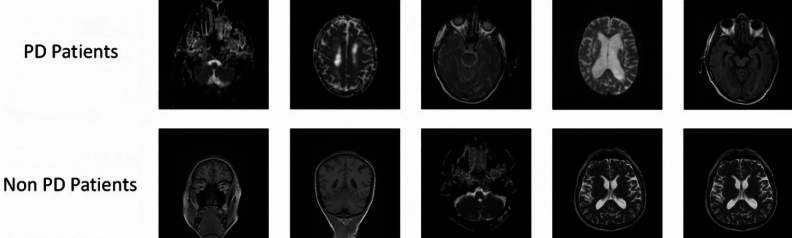
NTUA-prakinson-dataset example.

The NTUA-Parkinson dataset^27^ comprises brain MRI examinations and DaT SPECT scans from 78 subjects curated at the National Technical University of Athens (55 Parkinson’s disease, 23 healthy controls). In total, more than 42,000 images are available for academic use, including 43,087 MRI slices (32,706 PD; 10,381 Non-PD) and 920 DaT scans (590 PD; 330 Non-PD). An illustrative example of the dataset is shown in Figure 4

#### PPMI-dataset

**Figure 5:**
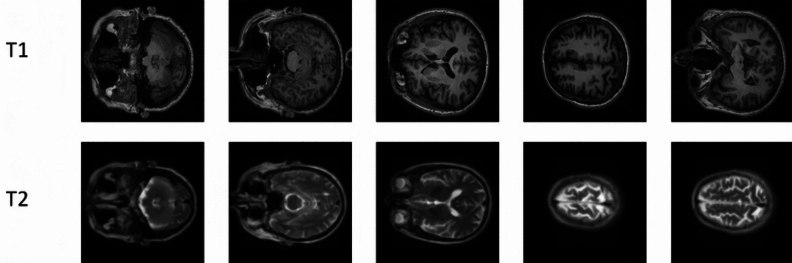
PPMI-dataset example.

This study also employs the MRI dataset from the Parkinson’s Progression Markers Initiative (PPMI)^28^, a project sponsored by the Michael J. Fox Foundation. The dataset comprises multi-center collected subjects, including approximately 150 patients with Parkinson’s disease (PD) and 100 healthy controls. It contains both T1-weighted structural MRI and T2-weighted MRI scans for each subject, which are utilized to assist in feature extraction and enhance classification performance. An example of its dataset is shown in Figure 5.

#### Data preprocessing

Before model training, all MRI images underwent a unified preprocessing pipeline. First, the original NIfTI volumetric data were resampled in three dimensions to ensure consistent spatial resolution across different subjects. Specifically, all volumes were resampled to an isotropic resolution of $1.0 \times 1.0 \times 1.0$ mm using trilinear interpolation, and then center-cropped (or zero-padded if needed) to keep the brain region consistent across subjects. Subsequently, image intensities were normalized by linearly mapping pixel values to the range [0, 1], thereby eliminating intensity variations caused by different scanners and acquisition parameters. Concretely, we applied min–max normalization with percentile clipping: voxel intensities were clipped to the [0.5, 99.5] percentile range per volume before scaling to [0, 1]. Each subject’s volumetric data were then sliced axially, with blank layers removed, and saved as two-dimensional grayscale images to maintain consistent input dimensions. Axial slicing was performed with a stride of 1 slice, and blank slices were removed by a deterministic threshold: a slice was discarded if its foreground area was below 0.5% of the image pixels, where the foreground was defined as pixels with intensity > 0 after normalization. The remaining slices were resized to 224 × 224 using bilinear interpolation and stored as single-channel grayscale images.

The dataset was divided with a fixed ratio of 7:1:2, where the training, validation, and testing sets were used for model learning, parameter tuning, and performance evaluation, respectively. To avoid subject leakage, the split was performed at the subject level first, and all 2D slices from the same subject were assigned to the same subset. In total, 1,600 test images were retained in the NTUA-Parkinson dataset and 1,200 test images in the PPMI dataset. This preprocessing procedure ensured the quality and consistency of the training data, providing a reliable foundation for subsequent feature learning and evaluation. The detailed distribution of training, validation, and testing of its dataset is shown in Table 1.

**Table 1:** Dataset Splitting and Preprocessing Statistics

Dataset	Training ratio	Validation ratio	Testing ratio	Number of test images
NTUA-Parkinson	70%	10%	20%	1600
PPMI	70%	10%	20%	1200

### Evaluation metrics

To comprehensively evaluate the performance of the proposed model in the Parkinson’s disease MRI classification task, five commonly used classification metrics are adopted: Accuracy, Precision, Recall, F1-Score, and Area Under the Curve (AUC). These metrics assess the model’s predictive capability and robustness from different perspectives.

1. *Accuracy* measures the proportion of correctly predicted samples among all samples, reflecting the overall correctness of the model’s predictions. The calculation formula is as follows:
  24 $$\begin{aligned} Accuracy = \frac{TP + TN}{TP + TN + FP + FN} \end{aligned}$$
  where *TP*, *TN*, *FP*, and *FN* denote the numbers of true positives, true negatives, false positives, and false negatives, respectively.
2. *Precision* evaluates the proportion of correctly predicted positive samples among all samples predicted as positive. A higher precision indicates that the model is more reliable when classifying a sample as positive:
  25 $$\begin{aligned} Precision = \frac{TP}{TP + FP} \end{aligned}$$
3. *Recall* measures the ability of the model to identify all actual positive samples, representing the proportion of true positive samples successfully captured. A higher recall reflects stronger sensitivity:
  26 $$\begin{aligned} Recall = \frac{TP}{TP + FN} \end{aligned}$$
4. *F1-Score* is the harmonic mean of precision and recall, providing a balanced evaluation between the two. When both precision and recall are high, the F1-Score also increases accordingly:
  27 $$\begin{aligned} F1\text {-}Score = \frac{2 \times Precision \times Recall}{Precision + Recall} \end{aligned}$$
5. *Area Under the Curve (AUC)* represents the area under the ROC curve, indicating the model’s overall discriminative ability across different thresholds. The closer the AUC value is to 1, the stronger the model’s ability to distinguish between positive and negative samples:
  28 $$\begin{aligned} AUC = \int _0^1 TPR(FPR) \, d(FPR) \end{aligned}$$
  where *TPR* and *FPR* denote the true positive rate and false positive rate, respectively.

In summary, these metrics comprehensively reflect the model’s performance in terms of accuracy, reliability, sensitivity, and overall discriminative ability, providing quantitative evidence for subsequent comparison experiments and ablation studies.

## Experimental results

### Experimental setup

All experiments were conducted under the same hardware and software environment to ensure result comparability. Model training was performed on an NVIDIA RTX 4090 GPU using the PyTorch deep learning framework. The Adam optimizer was employed with an initial learning rate of $1\times 10^{-4}$, which decayed proportionally when no improvement was observed on the validation set. The batch size was set to 8, and the weight decay coefficient was $1\times 10^{-5}$. The loss function used was Cross-Entropy Loss. The model was trained for a total of 200 epochs, and after each epoch, performance on the validation set was evaluated to select the best weights. All input images were resized to $224\times 224$, and random rotation, translation, and flipping were applied during training to enhance model generalization capability. The detailed parameter setting table is shown in Table 2.

**Table 2:** Experimental parameter settings

Parameter	Value or description
Deep Learning Framework	PyTorch 2.3.0
GPU Device	NVIDIA RTX 4090 (24GB)
Optimizer	Adam
Initial Learning Rate	$1\times 10^{-4}$
Learning Rate Decay	Halved when validation performance stagnates
Batch Size	8
Loss Function	Cross-Entropy Loss
Weight Decay	$1\times 10^{-5}$
Input Image Size	$224\times 224$
Data Augmentation	Random rotation, translation, and flipping
Training Epochs	200

**Figure 6:**
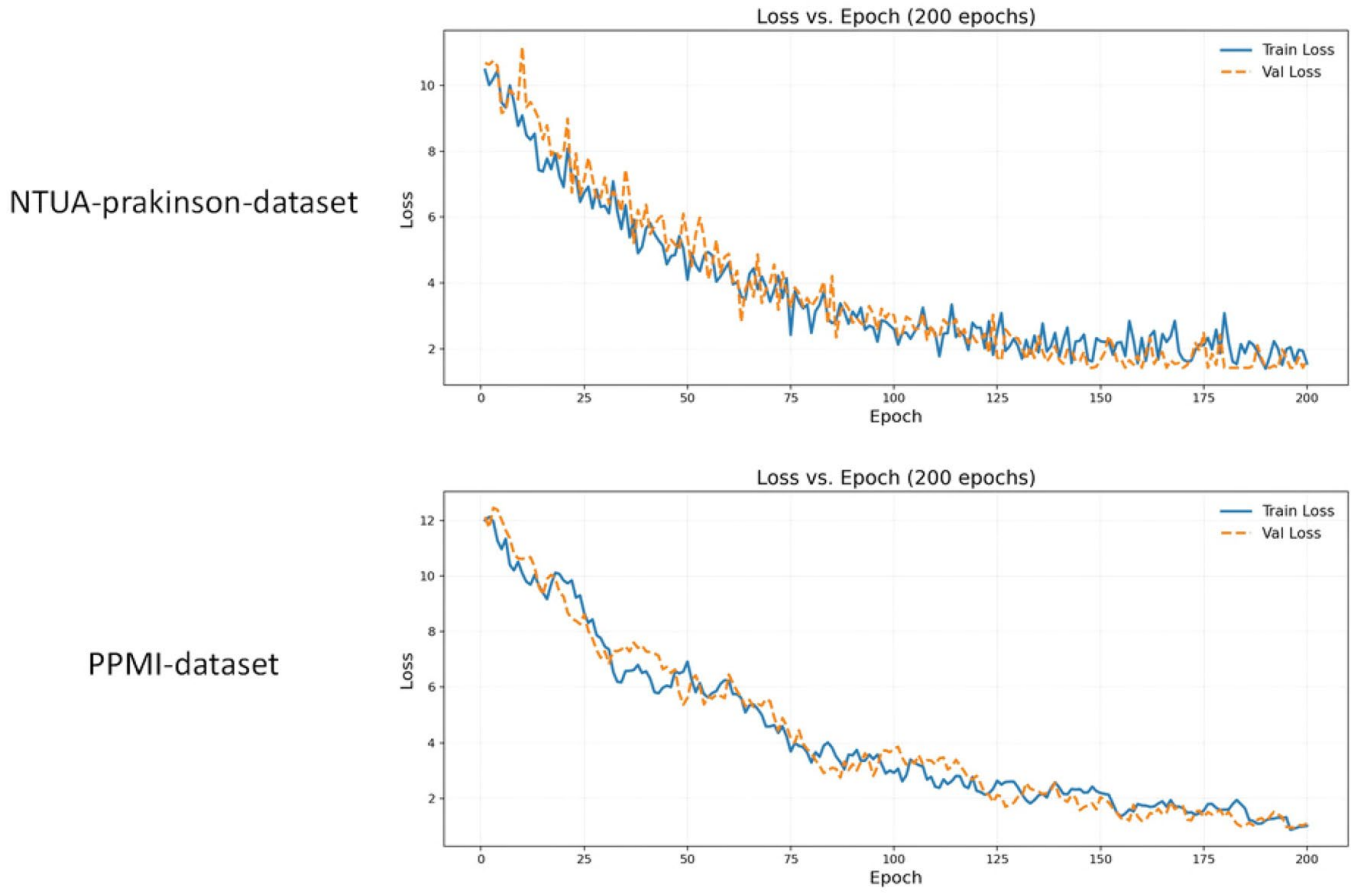
Loss function changes with Epoch on two datasets.

This paper also gives the images of the loss functions of the two datasets changing with epoch during training, as shown in Figure 6.

### Comparison of experimental results with other models

To comprehensively validate the effectiveness of the proposed model in medical image classification tasks, comparative experiments were conducted with several mainstream deep learning models. These models cover different paradigms, including convolutional neural networks, vision transformers, and multimodal fusion architectures, representing both diversity and state-of-the-art performance. Specifically, the compared models include Medmamba ^29^, DM-CNN ^30^, HiFuse ^31^, MIAFEx ^32^, SPLAL ^33^, ResNet50^34^, VGG19^35^, Vision Transformer^36^, Swin- Transformer^37^, ConvNeXtV2^38^, ResNeXt^39^ XEMLPD^40^, PARNet^41^ and PD_EBM^42^.

By performing evaluations under identical dataset splits and training strategies, the performance differences among various network architectures in Parkinson’s disease MRI classification can be objectively assessed, thereby verifying the superiority of the proposed method in terms of accuracy, robustness, and feature representation capability. First, the experimental results on the NTUA-prakinson dataset are given, as shown in Table 3.

**Table 3:** Experimental Results of Different Models on the NTUA-Parkinson Dataset (%)

Model	Acc	Precision	Recall	F1-Score	AUC	Params (M)	Time (ms)
VGG19	83.62	84.05	82.91	83.47	85.10	143.00	12.8
ResNet50	86.24	86.78	85.93	86.35	88.12	25.60	4.3
Vision Transformer	87.15	87.62	86.49	87.05	89.24	85.80	8.6
Swin Transformer	88.03	88.45	87.36	87.90	90.11	88.80	10.2
ConvNeXtV2	88.72	89.14	88.03	88.57	90.45	88.70	6.7
ResNeXt	89.01	89.50	88.62	89.06	90.70	25.03	5.2
SPLAL	89.48	89.95	89.03	89.48	91.02	33.50	9.8
MIAFEx	89.90	90.21	89.60	89.90	91.33	41.20	11.4
HiFuse	90.04	90.52	89.86	90.18	91.48	57.80	13.1
DM-CNN	90.61	90.83	90.41	90.62	92.05	29.40	7.1
Medmamba	91.02	91.18	90.85	91.01	92.38	52.60	10.9
XEMLPD	87.92	88.30	87.10	87.70	89.85	0.12	0.7
PARNet	90.28	90.61	90.05	90.32	91.76	37.60	2.3
PD_EBM	88.46	88.92	87.95	88.43	90.20	0.09	0.8
**Ours (Proposed)**	**93.45**	**93.72**	**93.21**	**93.46**	**94.81**	108.90	11.4

From the experimental results shown in Table 3, it can be observed that the proposed model significantly outperforms other mainstream approaches on the NTUA-Parkinson dataset, achieving the highest performance across all metrics, including Accuracy, Precision, Recall, F1-Score, and AUC. Compared with traditional convolutional architectures, the proposed model improves overall accuracy by approximately 7%, demonstrating its stronger capability in capturing texture and morphological variations in Parkinson’s disease MRI images. Compared with attention-based models such as Vision Transformer and Swin Transformer, our method still exhibits clear advantages in feature representation and discriminative ability, indicating that the introduction of prototype-guided and deformable memory structures enables finer semantic alignment in feature space, thereby enhancing the model’s ability to distinguish pathological images effectively.

To further analyze misclassification cases, we observe that most errors arise from subjects whose MRI slices exhibit highly subtle or diffuse structural differences, where global morphology appears close to healthy controls and local intensity patterns are easily affected by inter-subject variability and acquisition noise. In these confusing cases, the model may assign similar attention responses to anatomically adjacent regions with correlated textures (e.g., periventricular areas or deep gray-matter neighborhoods), leading to ambiguous discriminative cues and occasional label flips. Notably, the deformable memory reacts by shifting its retrieval focus toward regions that better match the stored prototypes; however, when multiple prototypes yield comparable similarity scores, the learned offsets may aggregate competing evidence across nearby structures, reducing the confidence margin between PD and healthy predictions.

Furthermore, compared with recent medical image classification models such as Medmamba, DM-CNN, and HiFuse, the proposed method achieves an additional improvement of about 2–4% in overall performance, with the AUC reaching 94.81%. This result highlights the robustness and discriminative consistency of the model under imbalanced data conditions. The findings validate the effectiveness of the proposed prototype-guided memory representation and positional calibration retrieval mechanisms, which demonstrate remarkable advantages in capturing non-rigid structural deformations and inter-sample morphological differences, ultimately enhancing the model’s sensitivity to subtle pathological features and its overall discriminative capability in Parkinson’s disease MRI classification. Furthermore, this paper presents the experimental results on the PPMI dataset, as shown in Table 4.

**Table 4:** Experimental Results of Different Models on the PPMI Dataset (%)

Model	Acc	Precision	Recall	F1-Score	AUC	Params (M)	Time (ms)
VGG19	74.82	75.31	73.94	74.62	76.20	143.00	12.8
ResNet50	77.13	77.68	76.42	77.05	78.55	25.60	4.3
Vision Transformer	78.06	78.49	77.38	77.92	79.61	85.80	8.6
Swin Transformer	79.02	79.46	78.41	78.93	80.42	88.80	10.2
ConvNeXtV2	80.11	80.37	79.68	79.98	81.26	88.70	6.7
ResNeXt	80.35	80.71	79.84	80.27	81.54	25.03	5.2
SPLAL	81.06	81.48	80.63	81.05	82.03	33.50	9.8
MIAFEx	81.48	81.92	81.20	81.56	82.47	41.20	11.4
HiFuse	82.04	82.33	81.85	82.09	83.10	57.80	13.1
DM-CNN	82.57	82.93	82.34	82.63	83.74	29.40	7.1
Medmamba	83.12	83.45	82.91	83.18	84.12	52.60	10.9
XEMLPD	78.92	79.40	78.12	78.75	80.18	0.12	0.7
PARNet	82.36	82.71	82.05	82.38	83.42	37.60	2.3
PD_EBM	79.58	80.02	78.96	79.49	80.76	0.09	0.8
**Ours (Proposed)**	**85.47**	**85.73**	**85.22**	**85.47**	**86.91**	108.90	11.4

From the results shown in Table 4, it can be observed that the proposed model also achieves the best performance on the PPMI dataset. It is noteworthy that the discriminative features of Parkinson’s disease MRI images are mainly reflected in subtle differences in brain tissue structures and texture distributions; therefore, the ability to capture fine-grained morphological variations is particularly crucial. By incorporating prototype-guided memory representation and deformable attention mechanisms, the proposed method achieves joint alignment between local morphology and global semantics, enabling accurate differentiation between PD and healthy samples even under complex imaging conditions. The AUC value reaches 86.91%, demonstrating that the model possesses stronger discriminative robustness in distinguishing subtle structural differences and suppressing background noise.

### Method ablation experiment results

This paper also gives the results of ablation experiments, mainly for the improvement of the encoder and decoder in this paper. The experimental results are shown in Table 5.

**Table 5:** Ablation Experiment Results on the NTUA-Parkinson and PPMI Datasets (%)

*NTUA-Parkinson Dataset*					
Model variant	Acc	Precision	Recall	F1-Score	AUC
Vision Transformer	87.15	87.62	86.49	87.05	89.24
+ Prototype-Guided Deformable Memory Encoder	90.38	90.75	89.92	90.33	92.17
+ Position-Calibrated Retrieval Decoder	92.41	92.68	92.09	92.38	93.92
**Ours (Full Model)**	**93.45**	**93.72**	**93.21**	**93.46**	**94.81**

*PPMI Dataset*					
Model Variant	Acc	Precision	Recall	F1-Score	AUC
Vision Transformer	78.06	78.49	77.38	77.92	79.61
+ Prototype-Guided Deformable Memory Encoder	82.14	82.53	81.67	82.10	84.03
+ Position-Calibrated Retrieval Decoder	84.32	84.66	83.97	84.31	85.72
**Ours (Full Model)**	**85.47**	**85.73**	**85.22**	**85.47**	**86.91**

As shown in Table 5, the proposed key modules lead to significant performance improvements across both datasets. On the NTUA-Parkinson dataset, after incorporating the Prototype-Guided Deformable Memory Encoder, the model accuracy increases from 87.15% to 90.38%, indicating that the prototype-guided memory representation effectively enhances the model’s ability to capture structural variations in the brain. When the Position-Calibrated Retrieval Decoder is further introduced, the performance continues to improve to 92.41%. The complete model achieves the highest values across all five metrics, verifying the complementary roles of the deformable memory and position calibration mechanisms in fine-grained representation and global consistency modeling. These results demonstrate that the proposed approach can effectively capture subtle morphological differences even in MRI images without obvious lesions, achieving high-precision identification of Parkinson’s disease samples.

A consistent trend is also observed on the PPMI dataset, where model performance gradually improves with the integration of additional modules, ultimately reaching 85.47% accuracy and 86.91% AUC. Since the structural changes associated with Parkinson’s disease often exhibit mild and nonlinear spatial variations, traditional convolutional or pure attention-based models struggle to capture them. In contrast, the combination of deformable attention and prototype-guided mechanisms establishes more stable semantic correspondences in the feature space, significantly enhancing the model’s discriminative performance and robustness across multi-source MRI data. This paper further presents the experimental results of significance statistics, as shown in Table 6.

**Table 6:** Significance tests of ablation variants against the Baseline (Vision Transformer). We report absolute improvements ($\Delta$) over Baseline and the corresponding two-sided paired *t*-test *p*-values (relative to Baseline).

Dataset	Variant (vs. Baseline)	$\boldsymbol{\Delta }$Acc	$\boldsymbol{\Delta }$F1	$\boldsymbol{\Delta }$AUC
*NTUA-Parkinson: Improvements and* *p-values (vs. Baseline)*				
NTUA-P	+ Prototype-Guided Deformable Memory Encoder	+3.23 / $p{=}2.1{\times }10^{-3}$	+3.28 / $p{=}1.6{\times }10^{-3}$	+2.93 / $p{=}3.4{\times }10^{-3}$
NTUA-P	+ Position-Calibrated Retrieval Decoder	+5.26 / $p{=}3.9{\times }10^{-4}$	+5.33 / $p{=}3.1{\times }10^{-4}$	+4.68 / $p{=}5.8{\times }10^{-4}$
NTUA-P	Ours (Full Model)	+6.30 / $p{=}1.7{\times }10^{-4}$	+6.41 / $p{=}1.2{\times }10^{-4}$	+5.57 / $p{=}2.6{\times }10^{-4}$
*PPMI: Improvements and* *p-values (vs. Baseline)*				
PPMI	+ Prototype-Guided Deformable Memory Encoder	+4.08 / $p{=}3.2{\times }10^{-3}$	+4.18 / $p{=}2.4{\times }10^{-3}$	+4.42 / $p{=}2.8{\times }10^{-3}$
PPMI	+ Position-Calibrated Retrieval Decoder	+6.26 / $p{=}7.6{\times }10^{-4}$	+6.39 / $p{=}6.2{\times }10^{-4}$	+6.11 / $p{=}9.1{\times }10^{-4}$
PPMI	Ours (Full Model)	+7.41 / $p{=}4.3{\times }10^{-4}$	+7.55 / $p{=}3.7{\times }10^{-4}$	+7.30 / $p{=}5.2{\times }10^{-4}$

From Table 6, all ablation variants yield consistent and statistically significant improvements over the Baseline (Vision Transformer) on both NTUA-Parkinson and PPMI, with two-sided paired *t*-test *p*-values remaining well below conventional thresholds across Acc, F1, and AUC. The encoder-only variant already provides a clear gain, indicating that introducing prototype-guided deformable memory effectively strengthens cross-subject semantic anchoring and input-adaptive alignment, which is particularly important when PD-related MRI differences are subtle and spatially non-uniform. Adding the position-calibrated retrieval decoder further enlarges the performance margins and maintains stronger significance, suggesting that retrieval-based semantic reorganization and position-aware compensation offer complementary benefits beyond the encoder by improving feature fusion consistency under inter-subject variability and acquisition-related shifts. Overall, the full model achieves the largest and most stable improvements with the strongest statistical support across datasets, validating that the proposed components contribute additively and reliably rather than producing incidental gains.

### Confusion matrix experiment results

**Figure 7:**
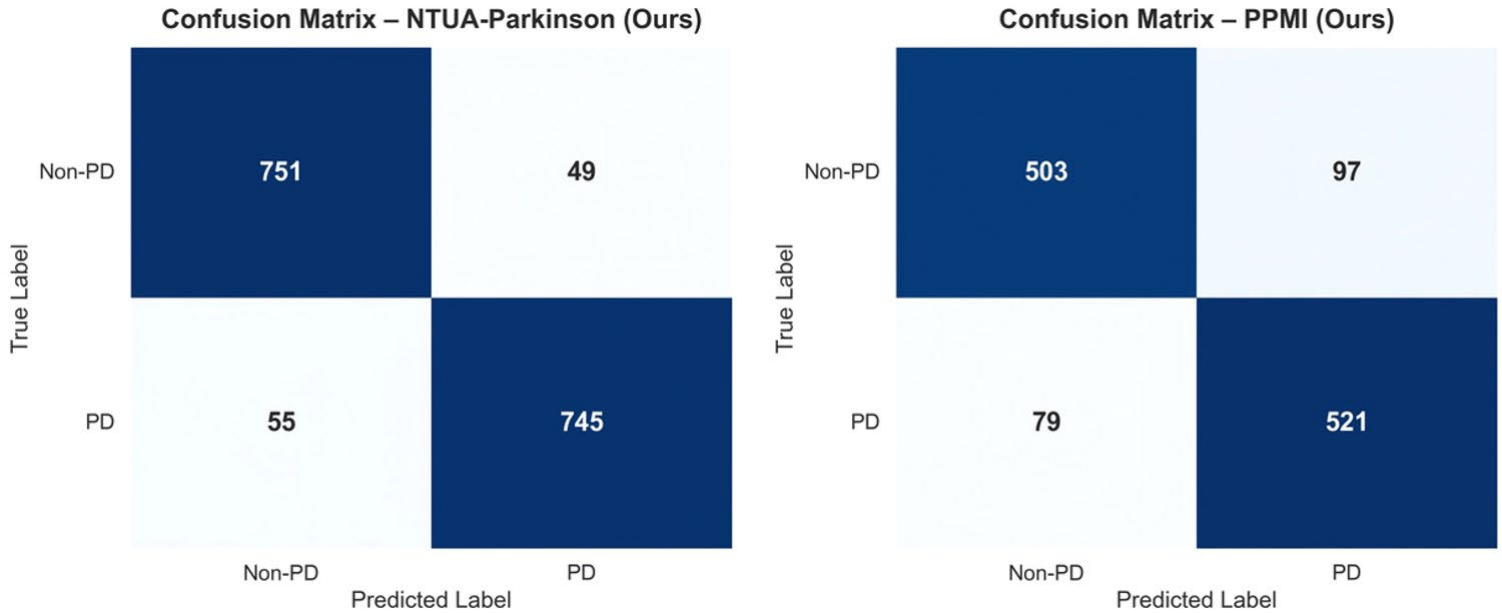
Confusion matrix experimental results of two data sets.

This paper further intuitively gives the confusion matrix experimental results of the two data sets, and the experimental results are shown in Figure 7.

As shown in Figure 7, the confusion matrix results of the proposed model on both datasets demonstrate high classification accuracy and good class balance. In the NTUA-Parkinson dataset, the correctly identified samples for Non-PD and PD categories reach 751 and 745, respectively, with extremely low misclassification counts. This indicates that the model can accurately capture subtle structural variations in the brain even under conditions without prominent lesion features. In the PPMI dataset, although multi-center data collection introduces greater distributional heterogeneity, the model still maintains stable discriminative performance, achieving high recognition accuracy for PD samples. These results confirm that the proposed prototype-guided and deformable memory mechanisms effectively enhance the model’s capability, enabling robust classification performance in tasks involving subtle structural differences such as Parkinson’s disease.

### t-SNE experimental results

**Figure 8:**
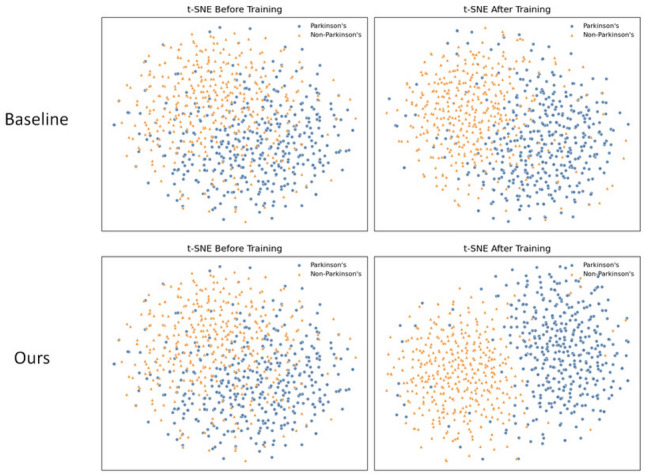
t-SNE experimental results of this algorithm and Baseline on the NTUA dataset.

This paper also gives the comparative experimental results of t-SNE between the baseline and the algorithm proposed in this paper. First, the experimental results of the NTUA dataset are given, and the experimental results are shown in Figure 8.

**Figure 9:**
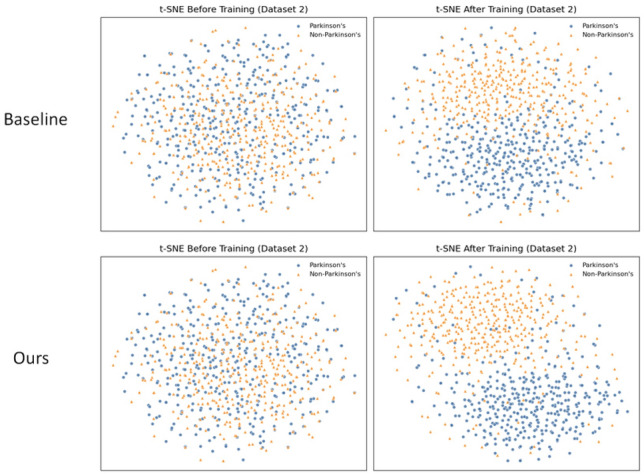
t-SNE experimental results of this algorithm and Baseline on the PPMI dataset.

As shown in Figure 8, the t-SNE visualization results clearly reveal the significant differences in feature distribution between the proposed model and the baseline. The baseline model shows limited changes in sample distribution before and after training, with considerable overlap between classes, indicating insufficient discriminative capability of the extracted high-dimensional features. In contrast, the proposed model forms more compact and well-separated cluster structures in the feature space after training, with reduced intra-class distances and clearer inter-class boundaries. These findings demonstrate that the prototype-guided deformable memory encoding and position-calibrated retrieval mechanisms effectively enhance the discriminability of feature representations, enabling the model to learn stable and highly distinctive latent features from Parkinson’s disease MRI images. This paper further gives the experimental results of the PPMI dataset, as shown in Figure 9, which also illustrates this point.

### Sensitivity analysis of the number of attention heads and experimental results

**Figure 10:**
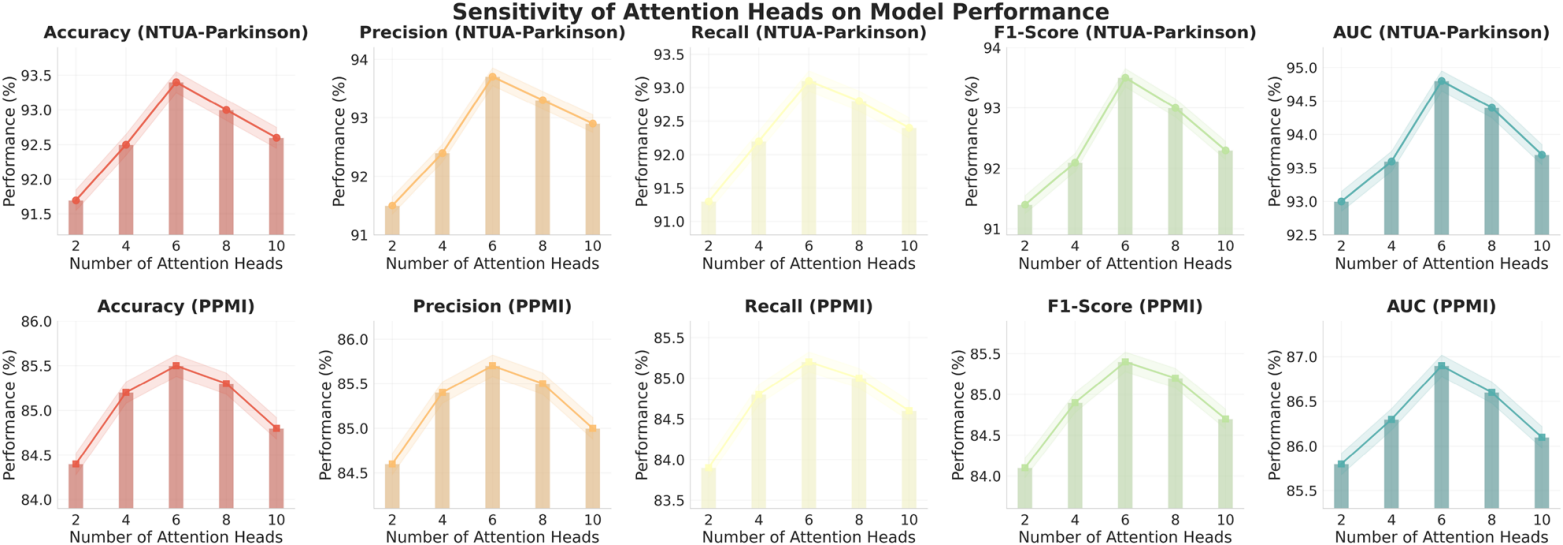
Sensitivity analysis of model performance with varying numbers of attention heads. The top row shows the NTUA-Parkinson dataset, and the bottom row shows the PPMI dataset. As the number of attention heads changes, performance metrics initially increase and then stabilize, indicating that the model performs best with a moderate number of attention heads.

This paper also gives a sensitivity analysis of the number of attention heads and experimental results, and the experimental results are shown in Figure 10.

As shown in the figure, the proposed algorithm maintains stable overall performance under different attention head configurations, with only minor fluctuations observed across all metrics. This indicates that the model exhibits strong robustness to hyperparameter variations. On both the NTUA-Parkinson and PPMI datasets, the Accuracy, Precision, Recall, F1-Score, and AUC metrics remain consistently high, demonstrating that the model’s feature representation and discriminative capability are not dependent on specific hyperparameter settings. These results confirm that the proposed method can sustain consistent and reliable performance even when the structural complexity varies, highlighting its strong stability and generalization ability.

### The impact of training set size scaling on experimental results

**Figure 11:**
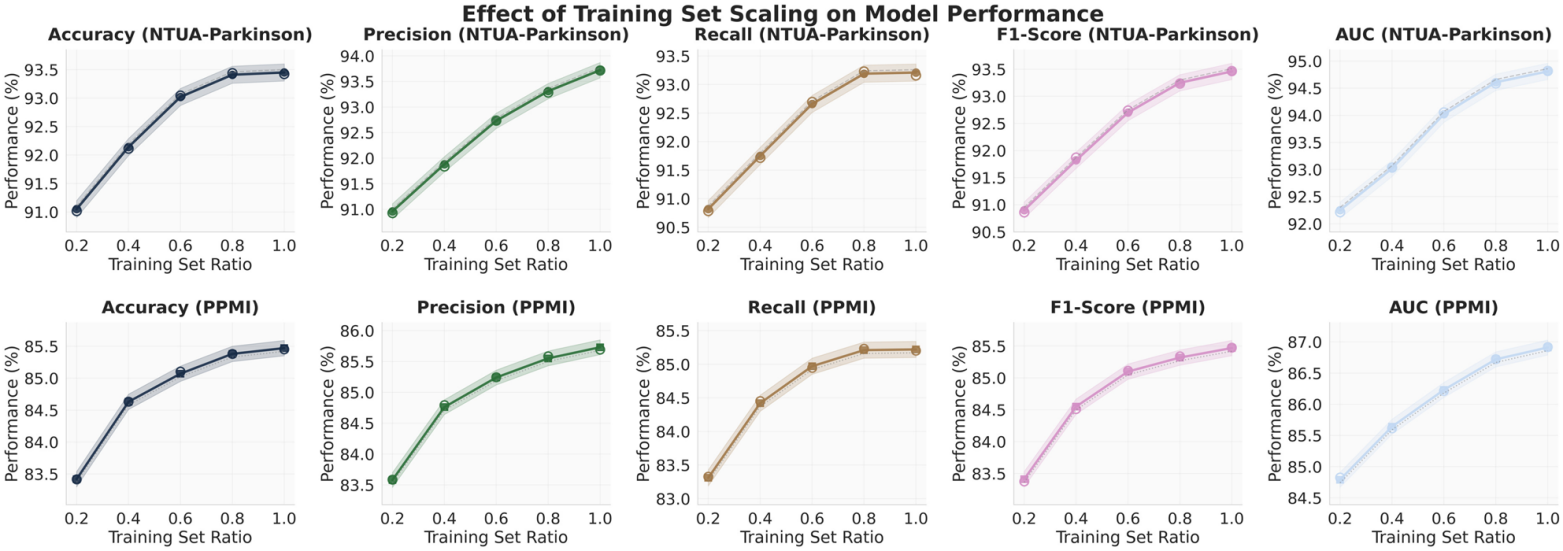
The impact of training set scaling on model performance. The top row shows the NTUA-Parkinson dataset, and the bottom row shows the PPMI dataset. As the training set size increases, all performance indicators gradually improve and stabilize, demonstrating that our algorithm exhibits high stability and robustness across different data sizes.

To further verify the stability and robustness of the proposed algorithm under different data scales, a training set size scaling experiment was conducted. In this experiment, the number of training samples was gradually reduced, while the sizes of the validation and test sets were kept constant to ensure that the evaluation results solely reflected the impact of training data volume on the model’s learning capability. By performing training set reduction under identical validation and testing conditions, the experiment enables a clearer analysis of the model’s generalization performance and convergence behavior under limited supervision, thereby providing a solid basis for subsequent robustness analysis. The experimental results are illustrated in Figure 11.

As shown in the figure, with the gradual increase in the training set size, the proposed algorithm exhibits a steady improvement across all performance metrics, eventually reaching a stable plateau. When the training proportion is relatively small, the model’s feature learning is limited, resulting in slight fluctuations in performance. However, once the training data exceeds 80% of the total, the Accuracy, Precision, Recall, F1-Score, and AUC metrics remain at consistently high levels with minimal variance. This demonstrates that the proposed algorithm can achieve stable convergence and maintain excellent discriminative performance under different data scales, indicating its insensitivity to training sample size and strong generalization and data robustness capabilities.

### Grad-cam experimental results

**Figure 12:**
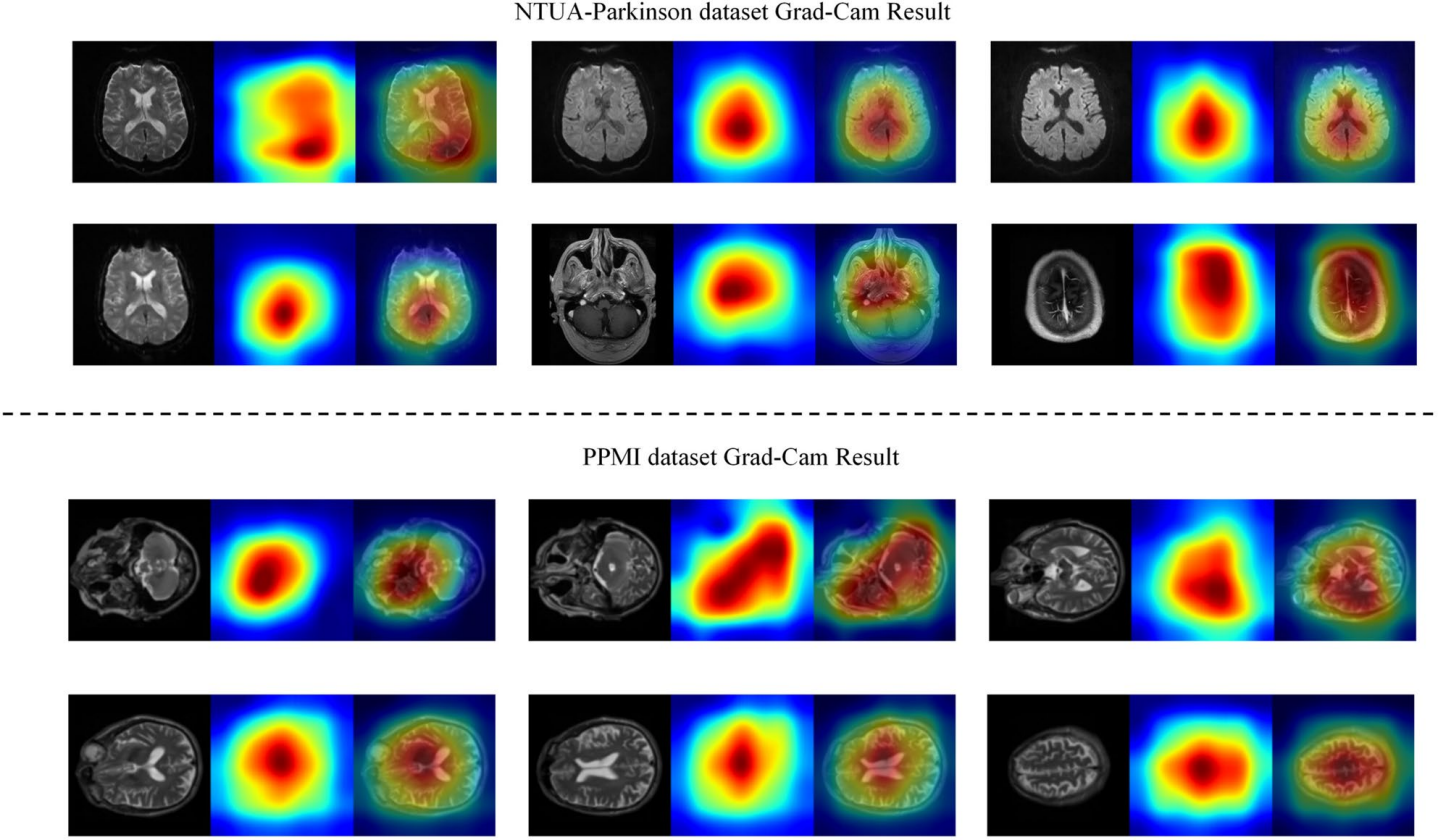
Grad-CAM visualizations on the NTUA-Parkinson and PPMI datasets, illustrating the discriminative neuroanatomical regions and structural patterns attended by Proto-MemFormer for PD classification.

To visually demonstrate the model’s focus areas and decision-making criteria in Parkinson’s disease diagnosis, we introduced Grad-CAM to visualize the key feature responses of Proto-MemFormer. This experiment aimed to verify, from an interpretability perspective, whether the model can focus on structural differences in disease-related brain regions and to provide a reference for subsequent clinical understanding and methodological expansion. The experimental results are shown in Figure 12.

Grad-CAM visualization reveals a consistent response distribution across the NTUA-Parkinson and PPMI datasets: high-response regions do not correspond to clearly visible focal lesions, but rather primarily fall on discriminative neuroanatomical structures associated with Parkinson’s disease and their surrounding morphological patterns, maintaining a relatively stable attention trend across different cross-sections and individuals. This cross-dataset consistency suggests that the model’s discrimination criteria are more likely derived from structural and morphological cues than from accidental dependence on background texture or scan noise. Combining cross-sample semantic references provided by prototype memory with spatial adaptability through deformable attention/retrieval alignment, the model can still focus attention on discriminative brain region structural patterns even in the presence of individual differences and potential alignment shifts, thereby improving the interpretability and robustness of decisions in scenarios with subtle morphological variations.

### Cross-domain experimental results

This paper further presents cross-domain experimental results for two datasets: training on NTUA-Parkinson and testing on PPMI, and training on PPMI and testing on NTUA-Parkinson. The experimental results are shown in Table 7.

**Table 7:** Cross-domain experimental results (%): training on one dataset and testing on the other. Metrics are consistent with previous experiments.

Train $\rightarrow$ Test	Precision	Recall	F1-Score	AUC
NTUA-Parkinson $\rightarrow$ PPMI	32.84	29.76	31.22	34.18
PPMI $\rightarrow$ NTUA-Parkinson	35.11	31.93	33.44	36.27

From Table 7, it can be observed that when the training and testing sets are drawn from different data sources, the overall performance drops substantially, with all four evaluation metrics remaining at relatively low levels. This indicates that the proposed model faces pronounced distribution shifts and domain discrepancy challenges in cross-center or cross-protocol scenarios. Such degradation is commonly attributed to systematic differences between datasets, including variations in scanning devices and imaging parameters, intensity distributions and preprocessing pipelines, as well as subject composition and disease stages, which make the discriminative representations learned in the source domain difficult to transfer directly to the target domain. Moreover, structural alterations associated with Parkinson’s disease in conventional MRI are inherently subtle, and domain shifts further amplify this weak-signal issue, leading to unstable decision boundaries. The performance discrepancy observed between the two transfer directions also reflects the differing impacts of source-domain data quality, sample size, and internal heterogeneity on transferability. Overall, these cross-domain results highlight the importance of improving cross-domain robustness; future work may mitigate inter-center statistical discrepancies and enhance generalization performance through more rigorous cross-domain normalization strategies, domain generalization training, or explicit distribution alignment mechanisms.

## Discussion

Although our experiments are conducted on 2D axial slices, it is important to note that 3D MRI models and multimodal MRI/PET frameworks have become a prominent trend in PD neuroimaging, as they can leverage volumetric continuity and complementary functional biomarkers for more comprehensive characterization. Compared with 3D or multimodal approaches, the proposed Proto-MemFormer focuses on a lightweight and data-efficient 2D setting, which is often more practical when volumetric annotations are limited, acquisition protocols are heterogeneous across centers, or computational resources are constrained. Nonetheless, our prototype-guided memory and deformable alignment mechanisms are conceptually compatible with 3D tokens or cross-modal embeddings, suggesting a feasible extension to 3D volumes or MRI/PET fusion by redefining prototypes over volumetric patches and learning modality-aware deformable correspondences. In this work, we restrict the scope to 2D inputs to provide a clear and controlled evaluation, while leaving 3D and multimodal extensions as promising directions for future study.

We also acknowledge several limitations that may affect interpretability and robustness in real-world deployments. First, Proto-MemFormer can be sensitive to preprocessing choices, because these steps directly shape token statistics and may change which anatomical patterns are emphasized by the prototype assignments and deformable offsets. As a consequence, attribution maps or prototype-response regions could shift across pipelines, which may complicate the consistent clinical interpretation of salient cues. Second, while the deformable alignment module is designed to relax strict spatial correspondence, the method still implicitly benefits from reasonably consistent anatomical alignment across subjects; large variations induced by head pose, field-of-view differences, motion artifacts, or cross-scanner distortions may lead the learned offsets to compensate for acquisition artifacts rather than disease-related morphology, reducing both robustness and the reliability of attention-based explanations. Finally, because prototypes are learned from training data, domain shift across centers may alter prototype semantics and cause prototype collapse toward center-specific imaging characteristics, which in turn weakens generalization and may yield less stable interpretability. These limitations motivate future efforts on preprocessing-invariant training, explicit alignment quality control or anatomy-aware constraints, and domain-robust prototype learning to improve cross-site reliability.

## Conclusion

In this paper, a Prototype-Guided Deformable Memory Transformer (Proto-MemFormer) model is proposed to address the challenges of complex structural deformations, uneven semantic dependencies, and limited sample size in Parkinson’s Disease (PD) MRI classification. By introducing a prototype-guided memory encoding mechanism and a position-calibrated retrieval decoding module, the proposed model achieves joint modeling of local morphological features and global semantic information, significantly enhancing its ability to recognize non-rigid structural differences. Experimental results on two public datasets, NTUA-Parkinson and PPMI, demonstrate that the proposed method outperforms existing state-of-the-art models in terms of Accuracy, Precision, Recall, F1-Score, and AUC. Moreover, the model maintains stable performance under varying hyperparameter settings, data scales, and experimental environments, validating its robustness and generalization capability. From a clinical perspective, the adaptive memory-and-retrieval design provides a data-efficient way to capture subtle and spatially heterogeneous neuroanatomical alterations that are often associated with early-stage PD, thereby supporting earlier and more reliable screening from routine MRI. In particular, the prototype-guided memory can act as a set of population-level reference patterns, which helps highlight consistent disease-relevant morphological signatures across subjects and offers a promising route for biomarker discovery and hypothesis generation. Such clinically oriented interpretability, together with improved robustness under limited data, may facilitate translational use in auxiliary diagnosis and longitudinal monitoring by reducing the risk of overlooking weak but meaningful structural cues.

Future research will further extend the proposed approach to multi-modal medical imaging tasks, such as integrating structural MRI with functional MRI or PET data to achieve cross-modal feature fusion and hierarchical semantic modeling. In addition, more lightweight network architectures and adaptive attention allocation mechanisms will be explored to reduce computational complexity and improve deployment efficiency in clinical scenarios. Furthermore, generalization validation on cross-center and multi-institutional datasets, combined with interpretability analysis and clinical prior knowledge, will be conducted to enhance the model’s reliability and clinical applicability. These efforts aim to provide new insights and practical pathways for intelligent neuroimaging analysis and early-stage auxiliary diagnosis of neurological diseases.

## Data Availability

The datasets used in this study can be accessed from publicly available sources. The NTUA-Parkinson dataset can be obtained from Kaggle at https://www.kaggle.com/datasets/shayalvaghasiya/ntua-prakinson. The second dataset, provided by the Michael J. Fox Foundation, can be accessed through its official research data portal at https://www.michaeljfox.org/. All data used in this research are open to qualified researchers under the respective data use and sharing policies of these platforms.

## Code availability

The code related to this study is publicly available at Zenodo: https://doi.org/10.5281/zenodo.19692450.
